# Alginate oligosaccharides attenuate obesity-associated kidney injury through modulation of the intestinal environment with renoprotection transferable by small intestinal contents

**DOI:** 10.3389/fnut.2026.1915255

**Published:** 2026-08-25

**Authors:** Zhijie Song, Kaiyuan Yang, Yuqing Cao, Zheng Yang, Xiaowei Yan, Dongxin Lu, Yong Zhao, Yanan Hao

**Affiliations:** 1 Shandong Provincial Key Laboratory of Brain Science, Institute of Brain Science and Brain-Inspired Research, Shandong First Medical University & Shandong Academy of Medical Sciences, Jinan, China; 2 School of Clinical and Basic Medicine, Institute of Basic Medicine, Shandong First Medical University & Shandong Academy of Medical Sciences, Jinan, China; 3 The School of Pharmacy, Shandong First Medical University & Shandong Academy of Medical Sciences, Jinan, China; 4 College of Life Sciences, Qingdao Agricultural University, Qingdao, China; 5 State Key Laboratory of Animal Nutrition, Institute of Animal Sciences, Chinese Academy of Agricultural Sciences, Beijing, China

**Keywords:** alginate oligosaccharide, gut-derived factors, high-fat diet, intestinal content transplantation, obesity-associated renal injury

## Abstract

**Background:**

High-fat diet (HFD)-induced obesity is closely associated with metabolic imbalance and kidney injury. Alginate oligosaccharides (AOS) are considered promising dietary bioactive compounds; however, whether they protect against obesity-associated renal damage has not been fully clarified. In this study, we investigated whether AOS ameliorates renal injury in HFD-fed mice and further used transplantation of small intestinal contents to explore the possible involvement of gut-derived factors.

**Methods:**

After AOS treatment, metabolic parameters, renal function, kidney histopathology, and related pathological alterations were evaluated. For the transplantation experiment, small intestinal contents collected from control or AOS-treated donor mice were administered to HFD-fed recipient mice, after which renal and metabolic phenotypes were assessed.

**Results:**

AOS improved HFD-induced metabolic disorders and alleviated renal injury. Mice treated with AOS showed lower serum blood urea nitrogen and creatinine levels, reduced renal lipid accumulation, and attenuated inflammatory and oxidative stress-related changes. Notably, recipient mice receiving small intestinal contents from AOS-treated donors also exhibited partial improvement in these abnormalities, whereas contents from control donors showed weaker or less consistent effects. These findings suggest that AOS-associated alterations in small intestinal contents may contribute to the observed renoprotective effects.

**Conclusion:**

AOS attenuated obesity-associated renal injury in HFD-fed mice, and this effect was at least partially transferable through transplantation of small intestinal contents. This study provides supportive evidence that gut-derived factors are involved, at least in part, in the renoprotective effects associated with AOS.

## Highlights

- Alginate oligosaccharides alleviated renal injury in high-fat diet-fed mice.
- Small intestinal content transplantation partially transferred the protective effects.
- Gut-derived factors may contribute to the renoprotective effects of AOS.
- AOS may improve obesity-associated renal injury through the gut–kidney axis.

## 1 Introduction

Kidney disease has become a major global public health concern, with a rapidly increasing prevalence that affects millions of people worldwide (1–3). Among the many factors involved in its pathogenesis, lipid abnormalities are now considered important contributors to the onset and progression of chronic kidney disease (CKD), largely through inflammation-related mechanisms (4–6). Excessive lipid deposition can lead to lipotoxicity, which in turn activates pro-inflammatory, fibrotic, and apoptotic pathways, ultimately resulting in cellular damage and renal dysfunction (7, 8). Chronic consumption of a high-fat diet (HFD), a major risk factor for obesity, further aggravates lipid accumulation and metabolic imbalance. Increasing evidence suggests that HFD-induced renal injury is driven by multiple pathological processes, including disordered renal lipid metabolism, aggravated dyslipidemia, persistent inflammation, and mitochondrial dysfunction (7, 9, 10). Given the growing burden of HFD-associated kidney injury, effective interventions are urgently needed.

Experimental models using HFD-fed mice reproduce several hallmark features of human metabolic syndrome, including dyslipidemia, insulin resistance, and progressive renal injury, and therefore provide a reliable system for mechanistic studies (11). Although a variety of therapeutic approaches have been explored for HFD-induced renal dysfunction, the clinical application of many pharmacological interventions remains constrained by high cost and potential side effects. These limitations underscore the need for safe, affordable, and effective alternative strategies, especially those aimed at correcting upstream metabolic disturbances.

Emerging evidence suggests that the gut plays an important role in the development of obesity-associated renal injury. High-fat diet feeding and obesity can disrupt intestinal homeostasis, reshape microbial composition and microbial metabolites, and impair intestinal barrier integrity. These changes may facilitate the translocation of pro-inflammatory and metabolically harmful factors into the circulation, thereby contributing to chronic low-grade inflammation, metabolic dysfunction, and renal injury. This gut-kidney interaction provides a mechanistic basis for exploring whether gut-derived factors are involved in the renoprotective effects of nutritional interventions. Alginate oligosaccharides (AOS), derived from the depolymerization of alginate, have attracted increasing attention as bioactive dietary carbohydrates because of their anti-inflammatory, antioxidant, and gut microbiota-modulating properties (12–14). Previous studies have suggested that AOS may improve metabolic disorders and intestinal homeostasis (15), indicating that AOS may have potential value as a nutritional intervention in obesity-related complications. Given the recognized involvement of inflammation, oxidative stress, and gut-derived factors in obesity-associated renal injury, AOS was selected in the present study as a candidate intervention to examine whether it could alleviate renal damage under high-fat diet conditions. Therefore, the present study aimed to investigate whether AOS could alleviate high-fat diet-induced renal injury in mice and to further explore the possible involvement of gut-derived factors by using small intestinal content transplantation. We hypothesized that AOS would improve renal injury under obese conditions and that intestinal contents shaped by AOS intervention might partially transfer these protective effects to recipient mice.

## 2 Materials and methods

### 2.1 Study design

The study was approved by the Institute of Animal Sciences, Chinese Academy of Agricultural Sciences (IAS2020-106) (15). All animal procedures were performed in accordance with the recommendations of the National Research Council Guide for the Care and Use of Laboratory Animals of the National Institutes of Health. Mice were housed at the Institute of Animal Science, Chinese Academy of Agricultural Sciences (Beijing, China) in a temperature-controlled environment with 50–70% humidity at 23 °C under a 12 h light/dark cycle (lights off from 8:00 p.m. to 8:00 a.m.). Three-week-old male ICR mice were used in all experiments.

The study consisted of two independent experiments. Experiment I was a donor experiment designed to generate sufficient small intestinal contents for transplantation. In this experiment, donor mice from the control and AOS-treated groups were maintained under the same dietary background, and the collected intestinal contents were pooled within each donor group. Experiment II was a recipient experiment designed to assess whether donor-derived intestinal contents could modify obesity-associated phenotypes in HFD-fed mice. All biochemical, histological, molecular, and metabolomic data presented in the figures were derived from recipient mice in Experiment II.

#### 2.1.1 Experiment I: preparation of small intestinal contents

Three-week-old male ICR mice were randomly assigned to two groups (30 mice/group): (1) Control (ddH2O) and (2) A10 (AOS, 10 mg/kg BW). Mice received either ddH2O or AOS at 10 mg/kg body weight by oral gavage (0.1 mL/mouse/day). After 3 weeks of treatment, all mice were maintained on a normal diet without further treatment for an additional 3 days. Small intestinal contents were then collected and used in Experiment II (15, 17).

#### 2.1.2 Experiment II: establishment of the obesity model and intestinal content transplantation

The small intestinal contents obtained in Experiment I were pooled within each donor group, diluted at a ratio of 1:1 in sterile 20% saline, and stored frozen for subsequent use. Before transplantation, the intestinal content suspension was prepared at 0.05 g/mL in sterile saline and filtered through a 70-μm cell strainer. Three-week-old male ICR mice were randomly divided into four groups (10 mice/group): (1) Control (normal diet + saline), (2) HFD [high-fat diet (Research Diet D12492, 60% fat) + saline], (3) A10-FMT [high-fat diet + transfer of small intestinal contents from AOS-treated mice in Experiment I], and (4) Con-FMT [high-fat diet + transfer of small intestinal contents from control mice in Experiment I]. For convenience, the transplantation groups are referred to as A10-FMT and Con-FMT throughout the manuscript, although the transferred material consisted of whole small intestinal contents rather than purified microbial communities. These groups were defined as non-obese, obese, A10-FMT-treated obese, and Con-FMT-treated obese mice, respectively. Recipient mice were not pretreated with antibiotics before intestinal content administration. This design was intended to evaluate the effects of donor-derived intestinal contents in HFD-fed mice with an intact endogenous microbiota (16–21).

**Figure 1:**
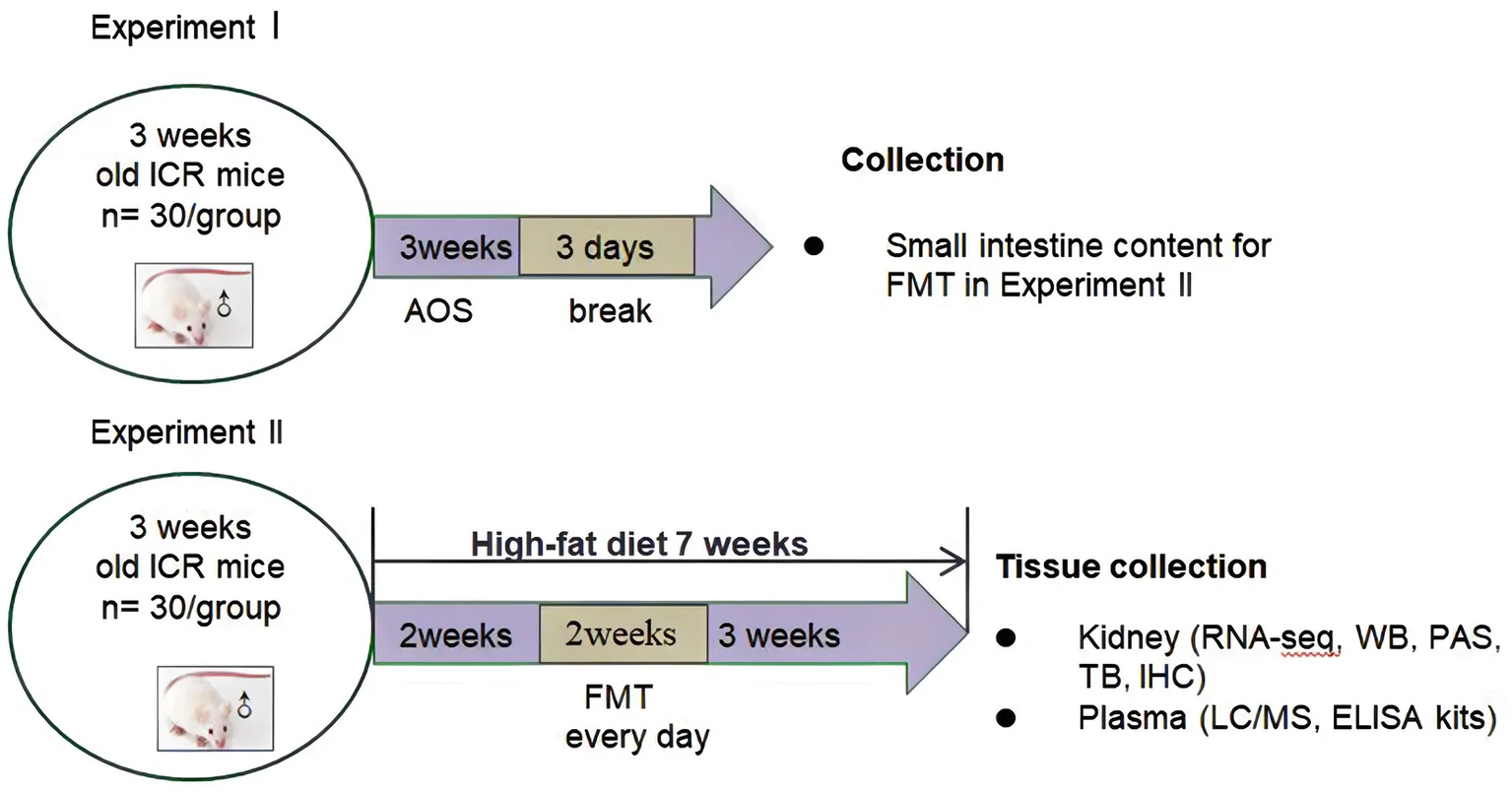
Experimental design of the present study. This figure was newly created for this present study however, part of the experimental design overlaps with our previously published work (15), published under a CC BY 4.0.

For the experimental procedure, mice were first fed either a normal diet or HFD for 2 weeks (from 3 to 5 weeks of age). They then received oral intestinal content inoculation once daily for 14 days. After transplantation, the mice continued on their respective diets until 10 weeks of age. At the end of the experiment, all mice were humanely euthanized, and samples were collected for subsequent analyses (Figure 1).

#### 2.1.3 Anesthesia and euthanasia

Mice were anesthetized with 4% isoflurane in an induction chamber. Anesthesia was maintained with 2% isoflurane (Cat. #: R510-22, RWD, Shenzhen, China) delivered via a nose cone. Mice were then euthanized by cervical dislocation to ensure a humane endpoint.

### 2.2 Biochemical examination

Blood samples were collected and centrifuged at 3000 rpm for 15 min to obtain serum, which was immediately stored at −80 °C until analysis. After thawing, the levels of triglycerides (TG), total cholesterol (TC), total antioxidant capacity (T-AOC), blood urea nitrogen (BUN), and creatinine (CRE) were measured using commercial assay kits from Nanjing Jiancheng Bioengineering Institute (Nanjing, China) according to the manufacturer’s instructions: TG (Cat. #: A110-1), TC (Cat. #: A111-1-1), T-AOC (Cat. #: A015-2-1), BUN (Cat. #: C013-2-1), and CRE (Cat. #: C011-2-1) (15, 22).

### 2.3 Gene expression analysis by RNA-seq and RT-qPCR

Transcriptomic analysis was performed as described in our previous studies (23, 24). Briefly, total RNA was extracted from kidney tissues using an RNA Isolation Kit (SA Biosciences, USA). RNA sequencing was conducted by BGI Genomics (Shenzhen, China) to obtain high-quality transcriptomic data. Differentially expressed genes were identified using the following comparisons: HFD vs. CON, A10-FMT vs. HFD, and Con-FMT vs. HFD. Gene Ontology (GO) and Kyoto Encyclopedia of Genes and Genomes (KEGG) enrichment analyses of the differentially expressed genes were subsequently performed using the BGI cloud platform.^1^ Candidate genes were selected from differentially expressed genes identified by transcriptomic analysis and further prioritized according to published evidence of involvement in renal physiology or kidney injury-related pathways. Then, RT-qPCR was performed under the following cycling conditions: 95 °C for 10 min; followed by 40 cycles of 95 °C for 15 s and 60 °C for 1 min; then dissociation curve analysis and cooling. The primers used in this study are listed in Table 1. At least three independent samples were analyzed.

**Table 1:** Primer sequences used for RT-qPCR analysis of selected genes.

Genes	Sequences (5'–3')	Fragment size (bp)	Accession No.
*β-Actin*	F: TCGTGGGCCGCTCTAGGCAC	255	NM_007393.3
	R: TGGCCTTAGGGTTCAGGGGGG		
*GAPDH*	F: AACAGCAACTCCCACTCTTC	111	NM_001289726.1
	R: CCTGTTGCTGTAGCCGTATT		
*AADAT*	F: GGATGTTGATGGGAGAGTCATC	92	NM_011834.2
	R: AGGTCTTAGGGCCAGTCATA		
*RENBP*	F: GCCTTGCTGCATCTCTTCTA	88	NM_001164704.1
	R: TCAGGTAGCCAAACCATTCC		
*AMY2A5*	F: GAAGTTCGTTCTGCTGCTTTC	99	NM_001042711.2
	R: CACTCGAACAGGTGGACAATA		
*POPDC3*	F: CATTTACCACCTCGCCAGTATC	90	NM_024286.1
	R: CCCAGCAAGCTGAAGACATAA		
*FDPS*	F: GGTATCAGAAGCCAGGCATAG	121	NM_001253751.1
	R: TCCAGCAGGTTCAGGTAGTA		
*HP*	F: CTGAGTATGGTGTGTACGTGAG	99	NM_001329965.1
	R: CATGTGCAGCCTTCTAGTGA		
*HSP90B1*	F: GAAGGAGTTCGGCACGAATA	95	NM_011631.1
	R: GGTGAGAAGACTGGAACCTAAG		
*YBX1*	F: GCAGCAGACCGTAACCATTA	113	NM_011732.2
	R: CTTTCCGATCCCTCGTTCTTT		
*HTR3A*	F: CTCGCTGAGACCATCTTCATT	102	NM_001099644.1
	R: ATCCAGGCTATTCTGTCTAGGA		
*KCNA1*	F: GAGCAGGAGGGAAATCAGAAG	100	NM_010595.3
	R: GGCGGGAGAGTTTGAAGATT		
*KCNMA1*	F: CAACGTGTTCTTCCTCCTCTAC	110	NM_001253358.1
	R: CAGGAGGGACTGTGAAGAAATC		
*SLC22A3*	F: CCAGGAAGCAGGATGAACAA	130	NM_011395.2
	R: CGGAGCTCAAGGGAAGAAATAG		
*LIPE*	F: GCCAACGGATACCGTAGTTT	88	NM_001039507.2
	R: TGCGGTTAGAAGCCACATAG		
*CALR*	F: TTCAGCCCTCATCTGGTTTC	96	NM_007591.3
	R: TCCTAGAGTTGGAGGGTAGTG		
*SLC29A4*	F: CCGTTCTGTCCCTTTCTTGT	105	NM_146257.2
	R: GGCCTGTGTATGAGGAATGAG		
*KLOTHO*	F: GCTGGTCTTCATCTCGTTTCT	111	NM_013823.2
	R: CCAGGCAGACGTTCACATTA		

### 2.4 Immunohistofluorescence (IHF) study

Kidney tissues were fixed in 4% paraformaldehyde for at least 4 h, dehydrated through a graded ethanol series, embedded in paraffin, and sectioned at 5 μm thickness for immunohistofluorescence staining (25). Briefly, sections were rehydrated and blocked with normal goat serum in TBS (15% BSA: goat serum: TBS = 2:1:7), followed by incubation overnight at 4 °C with primary antibodies diluted 1:150 in TBS containing 1% BSA. The antibodies used for histopathological analysis are listed in Table 2. After washing three times with TBST (TBS containing 0.1% Tween 20) for 10 min each, sections were incubated with fluorescent secondary antibodies (Beyotime Institute of Biotechnology, China) at 37 °C for at least 30 min. After three additional washes with TBST, the sections were counterstained with DAPI. Stained sections were observed using a Leica fluorescence microscope (Germany), and the images were analyzed using ImageJ software.

**Table 2:** Antibodies used for IHF.

Antibodies	Cat. No.	Company
*β-Actin*	D110001-0200	www.sangon.com.cn
*ANG II*	bs-0587R	www.bioss.com.cn
*AGT*	D260050	www.sangon.com.cn
*ATR1*	D220200	www.sangon.com.cn
*IL-22R*	bs-2624R	www.bioss.com.cn
*TGF-β1*	bsm-33345M	www.bioss.com.cn
*TNF-α*	bs-10802R	www.bioss.com.cn
*ZG16*	bs-55208R	www.bioss.com.cn
*CYP26β1*	bs-7538R	www.bioss.com.cn
*α-SMA*	bs-10196R	www.bioss.com.cn
*ZO-1*	ab-190085	Abcam
*Claudin 1*	ab-211737	Abcam
*Claudin 11*	bs-2183R	www.bioss.com.cn
*E-cadherin*	ab-11512	Abcam

### 2.5 Toluidine blue (TB) staining

Kidney tissues were fixed in 4% paraformaldehyde for at least 4 h, embedded in paraffin, sectioned at 5 μm, and subjected to toluidine blue (TB) staining. Briefly, sections were dewaxed, rehydrated to water, and stained with 0.1% TB solution (Shanghai Sangon Biotechnology Co., Ltd., China; Cat. #: E670105-0500) at room temperature for 5 min. Sections were then rinsed for 2 min, dehydrated through a graded ethanol series, and sealed with neutral resin for permanent preservation. The stained sections were examined using a Leica microscope (Germany).

### 2.6 Periodic acid-Schiff (PAS) staining

Kidney tissues were fixed in 4% paraformaldehyde for more than 4 h, dehydrated through a graded ethanol series, embedded in paraffin, and sectioned at 5 μm. PAS staining was then performed according to the manufacturer’s instructions using a commercial kit (Beijing Solarbio Technology Co., Ltd., China; Cat. #: G1281). Briefly, sections were dewaxed, rehydrated to ddH2O, and stained according to the kit protocol. After staining, the sections were sealed with neutral resin for permanent preservation. Images were acquired using a Leica microscope (Germany).

### 2.7 Metabolite quantification and statistical analysis

The metabolites highlighted in this study were compared based on their relative abundance in each group. Data are presented as relative metabolite levels, and group differences were evaluated using one-way ANOVA. A value of *p* < 0.05 was considered statistically significant.

### 2.8 Statistical analysis

Data are presented as mean ± SEM. Statistical analyses were performed using SPSS software (IBM, NY, USA). Differences among multiple groups were analyzed using one-way ANOVA followed by LSD *post hoc* multiple-comparison tests. All groups were compared with each other for each parameter. A value of *p* < 0.05 was considered statistically significant.

## 3 Results

### 3.1 A10-FMT improved renal function and pathology

**Figure 2:**
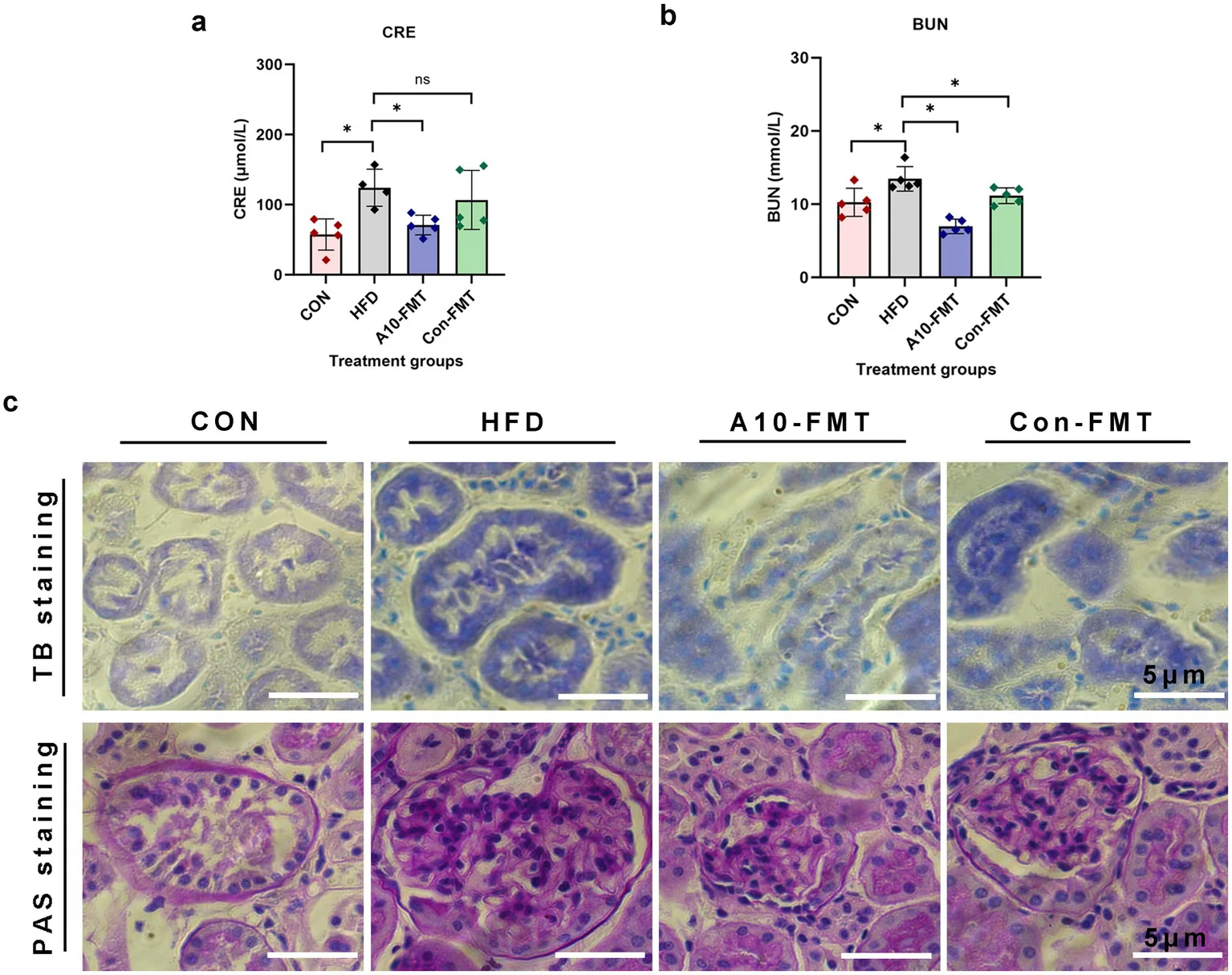
A10-FMT benefited HFD-injured kidney functions and structure. **(a,b)** Plasma creatinine (CRE) and blood urea nitrogen (BUN) levels. ^*^p < 0.05. **(c)** Representative TB and PAS staining of kidney sections. Scale bar = 5 μm.

Functional assessment of renal biomarkers demonstrated HFD-induced elevations in serum creatinine (CRE) and blood urea nitrogen (BUN) levels (26, 27), while A10-FMT significantly reduced both parameters (Figures 2a,b). Notably, Con-FMT produced a partial improvement in BUN (Figure 2b), whereas A10-FMT reduced both CRE and BUN, suggesting a stronger protective effect of intestinal contents derived from AOS-treated donors. Additionally, histological analysis across the four experimental groups (Con, HFD, A10-FMT, Con-FMT) revealed significant structural alterations. The TB and PAS staining showed that HFD-induced glomerular abnormalities, including expanded inter-glomerular spaces, thickened mesangial matrix and disrupted glomerular membrane architecture, which were visibly attenuated in the A10-FMT group (Figure 2c). TB staining showed enhanced blue staining in the HFD group compared with the Con group, suggesting abnormal tissue changes associated with HFD-induced renal injury, and these changes were improved by A10-FMT treatment (Figure 2c). The PAS staining corroborated these findings, revealing that HFD-induced hypercellularity (increased nuclear counts) was reversed by microbiota transplantation interventions. These results suggest that A10-FMT ameliorates renal pathology and function disrupted by HFD.

To further understand the protective role of A10-FMT on renal health, we conducted transcriptome analysis of kidney gene expression. PCA demonstrated distinct gene expression changes induced by HFD, A10-FMT, and Con-FMT compared to controls (Figure S1a). Functional enrichment via Metascape highlighted key biological processes affected by HFD and microbiota interventions, such as “monocarboxylic acid metabolic process,” “immune system process,” and “multicellular organismal process” (Figures S1b,c). Eighteen selected genes associated with renal function and injury-related pathways were validated by RT-qPCR to verify the transcriptome data (Figures S1d,e). Genes like LIPE involved in “aldosterone synthesis and secretion,” HTR3A, FDPS, KCNA1, and KCNMAL in “regulation of ion transport,” SLC22A3 and SLC29A4 in “kidney drug transport,” and others linked to “kidney cancer” and “electrolyte homeostasis” were examined (28–32). Notably, KLOTHO, recognized for its anti-aging and reno-protective roles by reducing oxidative stress and fibrosis, was upregulated by A10-FMT (22). POPDC3 was retained in the validation panel as a differentially expressed candidate gene from transcriptomic screening, but its specific role in obesity-associated renal injury remains to be clarified. This concordance between transcriptomic and RT-qPCR results substantiate the mechanistic insights.

### 3.2 A10-FMT was associated with alterations in blood metabolites and gut microbial profiles in HFD-fed mice

**Figure 3:**
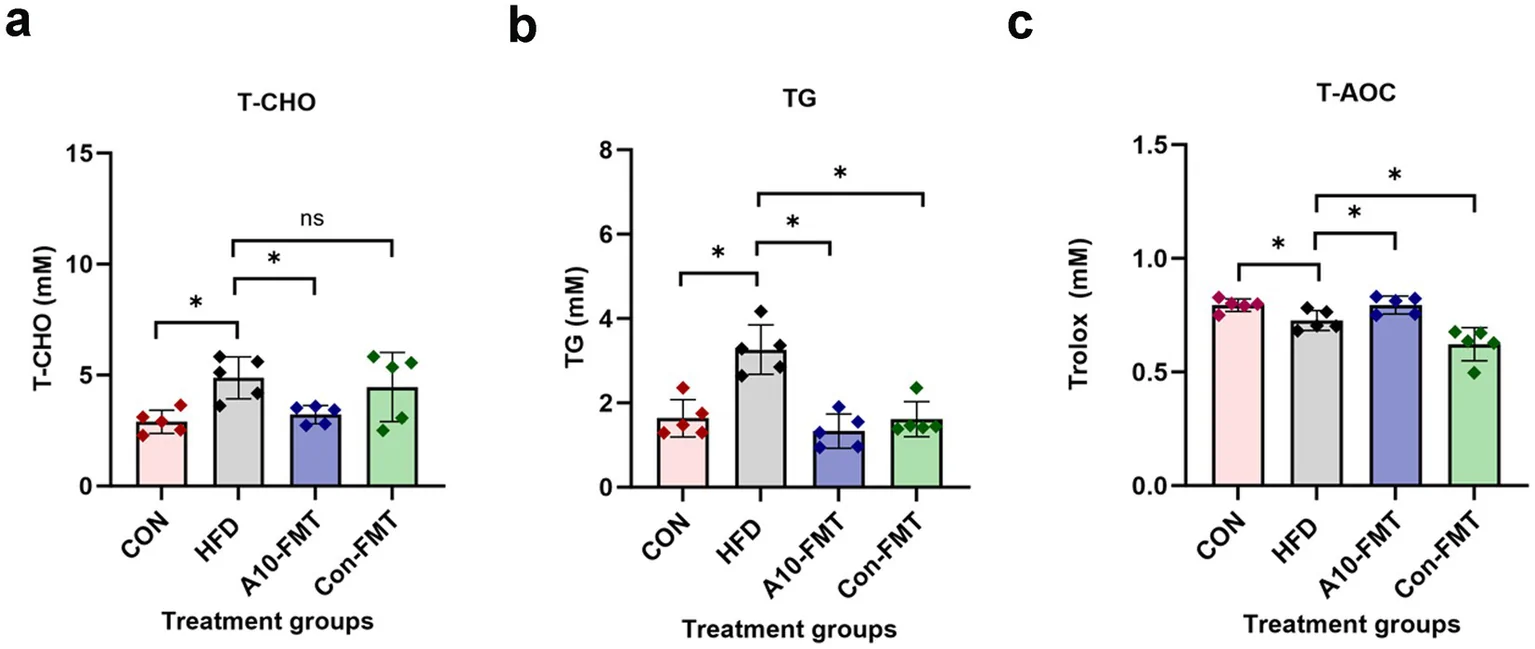
A10-FMT benefited blood metabolism. **(a)** TC levels in the CON, HFD, A10-FMT, and Con-FMT groups *p < 0.05. **(b)** TG levels in the CON, HFD, A10-FMT, and Con-FMT groups. ^*^p < 0.05; **(c)** T-AOC level in the C, HFD, A10-FMT, and Con-FMT groups ^*^p < 0.05.

HFD is known to induce obesity and increases blood lipid levels. We assessed blood lipid concentrations by testing total glycerides (TG) and total cholesterol (TC). HFD increased blood TG and TC levels compared to the Con, while both A10-FMT and Con-FMT reduced TG levels (Figures 3a,b). However, only A10-FMT decreased TC to levels comparable to the control group, while Con-FMT showed no significant effect (Figure 3b). In addition, antioxidant capacity in the blood was improved by A10-FMT and Con-FMT (Figure 3c). These data show that A10-FMT could ameliorate HFD-disrupted blood metabolome. LC–MS analysis showed that HFD altered blood metabolite profiles, decreasing compounds beneficial to the kidney and increasing harmful ones. A10-FMT reversed these changes, elevating beneficial metabolites such as *α*-tocotrienol (*p* < 0.05) and tetrahydro-deoxycorticosterone (*p* < 0.05) (Figure S2d), while reducing harmful metabolites like normetanephrine (*p* = 0.192) (Figure S2d). The parallel restoration of microbial function and metabolite profiles underscores A10-FMT’s dual action: rectification of gut ecological networks and mitigation of metabolic nephrotoxicity.

HFD also disrupts gut microbiota balance (7, 15, 33, 34). 16S rRNA analysis confirmed microbial imbalances (Figures S2b,c). Functional metagenomic predictions indicated HFD altered pathways related to membrane transport, carbohydrate metabolism, energy metabolism, and glycan biosynthesis, changes that A10-FMT reversed (Figure S2a). These findings indicate that intestinal content transfer from AOS-treated donors was associated with changes in both microbial composition and circulating metabolites in recipient mice.

### 3.3 A10-FMT was associated with altered renal RAS-related markers in HFD-fed mice

**Figure 4:**
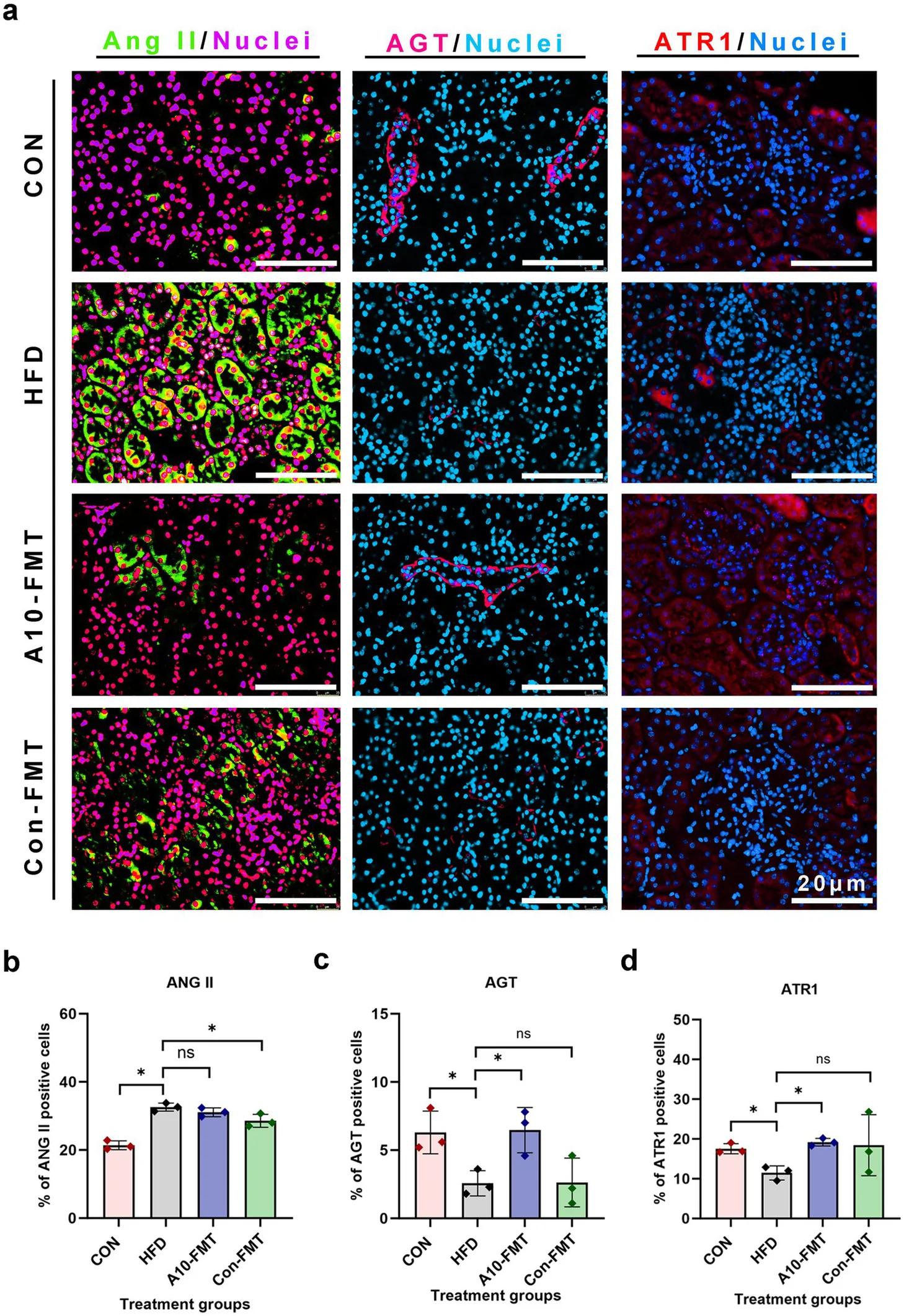
A10-FMT rescued kidney RAS dysfunction. **(a)** IHF investigation on RAS-related markers (ANG II, AGT, ATR1) expressions in the CON, HFD, A10-FMT, and Con-FMT groups. Scale bar: 20 μm. **(b–d)** Statistics analysis of immunofluorescence results. *p < 0.05.

HFD altered the expression of several renal RAS-related markers, including ANG II, AGT, and ATR1, and these changes were modulated after transplantation of intestinal contents from AOS-treated donors. The renin-angiotensin system (RAS), comprising renin, angiotensin-converting enzyme, and angiotensinogen, plays a pivotal role in regulating systemic blood pressure, electrolyte balance, and renal hemodynamics (35). In the current study, HFD significantly disturbed renal RAS homeostasis, elevating the protein level of ANG II in the kidney while A10-FMT rescued it (Figures 4a,b) (36). HFD reduced the protein levels of the angiotensinogen (AGT) and angiotensin type II receptor 1 (ATR1) in the kidney (Figures 4a,c,d), both related to renal functions (37, 38). Additionally, A10-FMT significantly restored the protein levels of AGT and ATR1, unlike Con-FMT. These findings demonstrate that A10-FMT intervention stabilizes RAS signaling integrity in the kidney, suggesting microbiota modulation is linked to improved renal tubular transport function.

### 3.4 A10-FMT restored the HFD-injured renal vascular development and metabolic functions

**Figure 5:**
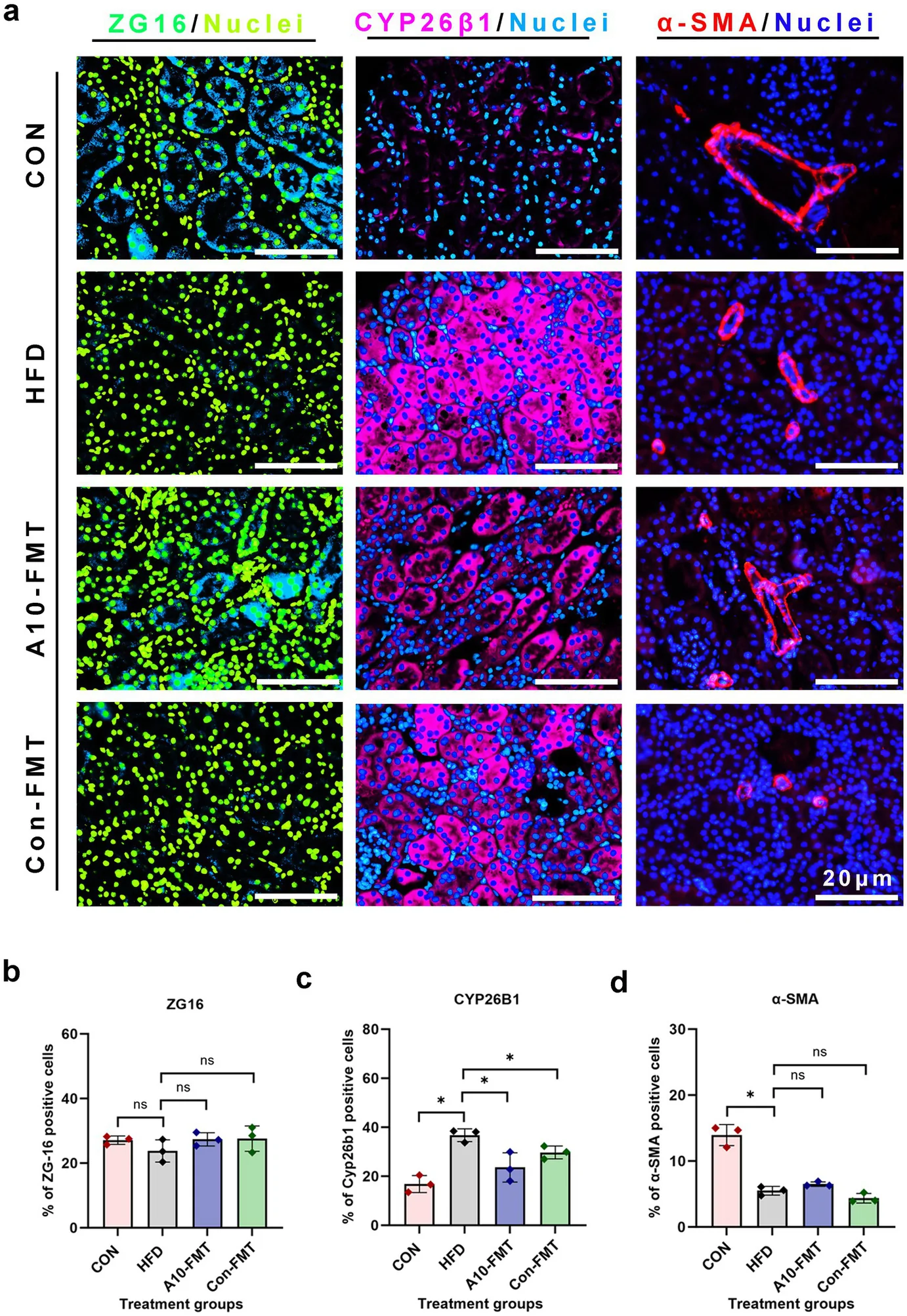
A10-FMT ameliorated kidney development. **(a)** IHF study of ZG16, CYP26β1, α-SMA expressions in the CON, HFD, A10-FMT, and Con-FMT groups. Scale bar: 20 μm. **(b–d)** Statistics analysis of immunofluorescence results. *p < 0.05.

Alpha-smooth muscle actin (α-SMA) is a critical mediator renal vascular development (22). HFD reduced α-SMA expression, which both A10-FMT and Con-FMT restored (Figures 5a,c), suggesting microbiota-dependent modulation of renal vascular integrity. Further analysis revealed that HFD downregulated zonulin granule protein 16 (ZG16), important for carbohydrate binding and xenobiotic transport (39). This suppression was reversed by A10-FMT treatment (Figures 5a,b), indicating enhanced metabolic detoxification capacity. Cytochrome P450 26B1 (CYP26B1) plays a vital role in iron ion binding and oxidoreductase activity involved in drug metabolism and cholesterol synthesis (40). HFD increased the protein level of CYP26B1, while A10-FMT decreased it significantly (Figures 5a,c), implicating microbiota-driven suppression of aberrant lipid synthesis pathways. These results indicate that A10-FMT’spotential to restore renal vascular development markers, enhance metabolic transport processes, and counteract HFD-induced dysregulation.

### 3.5 A10-FMT prevented kidney cellular inflammation

**Figure 6:**
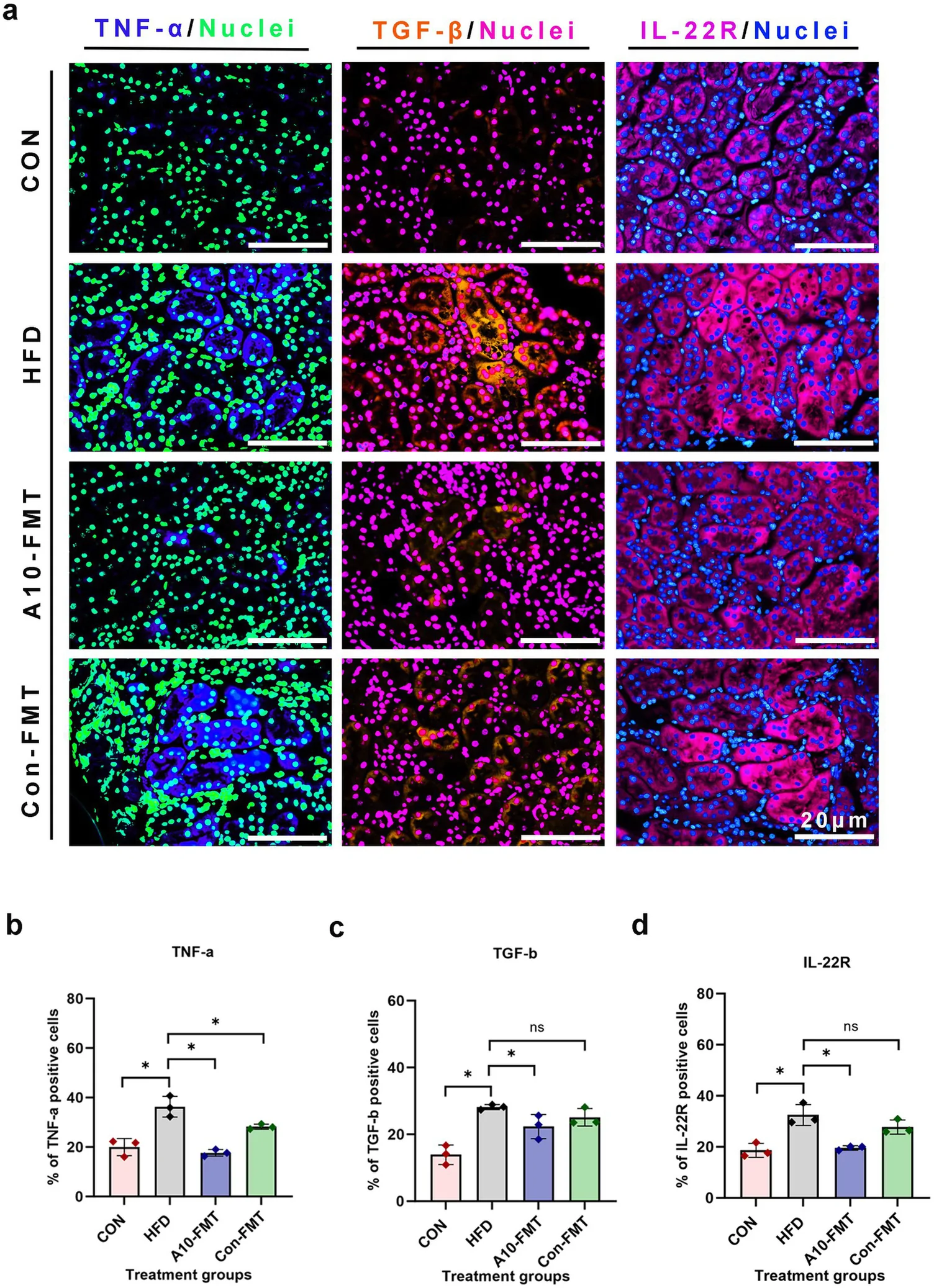
A10-FMT decreased kidney inflammatory reaction. **(a)** IHF investigation on inflammatory marker (TNF-α, TGF-β, IL-22R) in different groups. Scale bar: 20 μm. **(b–d)** Statistics analysis of immunofluorescence results. ^*^p < 0.05.

Based on the results of RNA-seq, there were changed genes enriched in the “immune system process.” Consistent with the previous study (10, 33, 41, 42), HFD could damage kidney function through multiple signal pathways, especially the inflammatory pathway. We examined immune-related protein levels by IHF. HFD increased TNF-α, TGF-β, and IL-22R levels linked to inflammation, as well as some metabolites (creatinine, urea, and uric acid activity) (43), all significantly reduced by A10-FMT (Figure 6). Con-FMT did not significantly decrease TGF-β and IL-22R (Figures 6a,c,d). The current study found that A10-FMT could protect the kidney from inflammatory reactions and cell damage. Therefore, the data signified that A10-FMT improved renal health by suppressing pro-inflammatory signaling cascades and mitigating of HFD-driven metabolic dysregulation. The concordance between transcriptomic immune pathway activation and biochemical validation showed microbiota modulation as a viable strategy to counteract diet-induced renal inflammation.

### 3.6 A10-FMT recovered tight junction protein levels in the kidney

**Figure 7:**
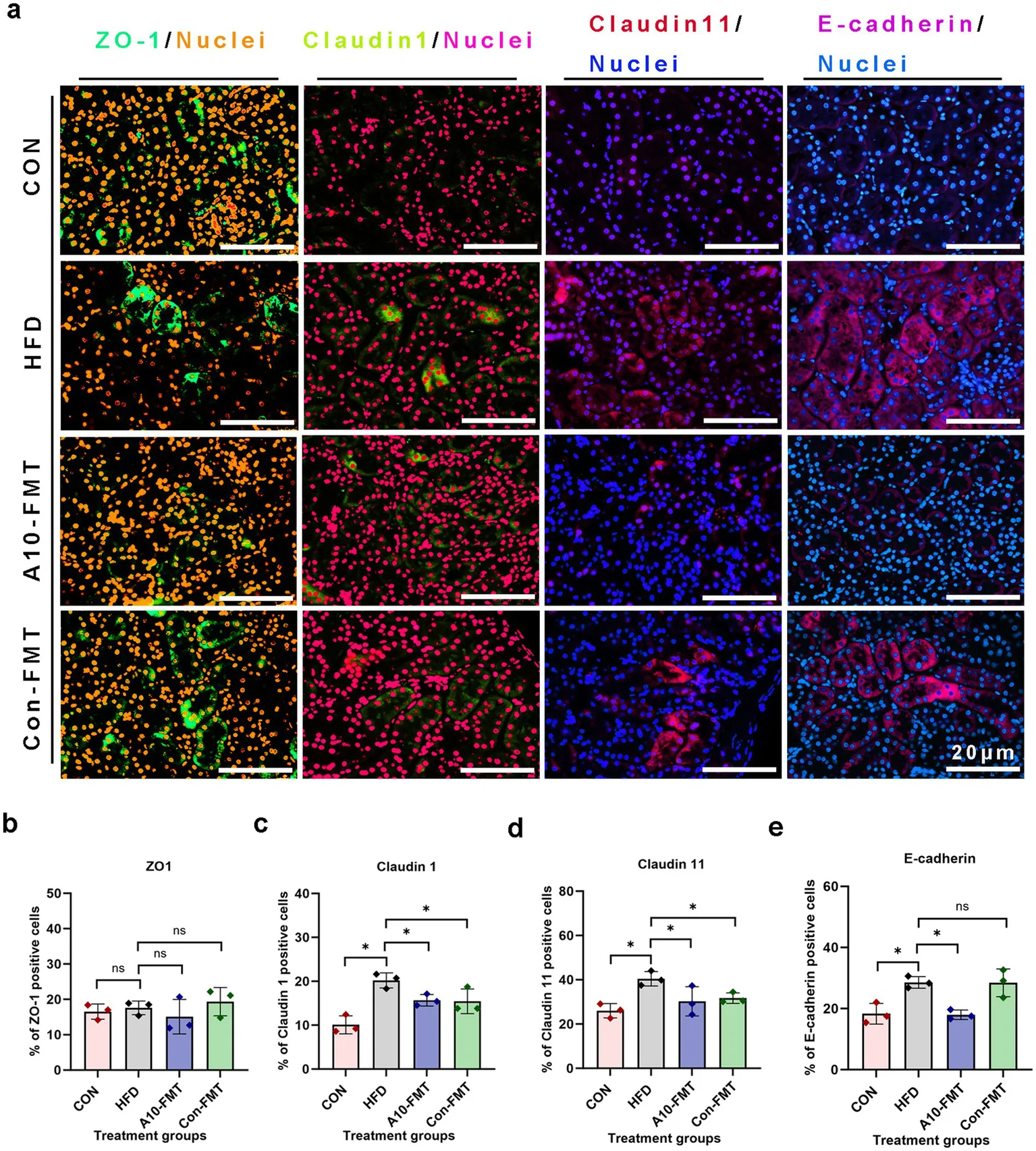
A10-FMT improved renal cellular tight junction. **(a)** IHF staining of ZO-1, Claudin 1, Claudin 11, E-cadherin expressions in the different treatments. Scale bar: 20 μm. **(b–e)** Statistics analysis of immunofluorescence results. ^*^p < 0.05.

It is known that the kidney is an essential organ with transportable functions to control the water and electrolyte balance and maintain acid–base balance. Herein, we assessed tight junction-associated proteins. ZO-1, Claudin 1, Claudin 11, and E-cadherin are crucial for renal function. The protein level of ZO-1 was increased in the HFD group, while A10-FMT decreased the protein level (Figures 7a,b). Similarly, Claudin 1, Claudin 11, and E-cadherin levels were dramatically increased in the HFD group and decreased by A10-FMT (Figures 7a,c–e). Different from A10-FMT, Con-FMT had no effect on E-cadherin levels (Figures 7a,e). These results suggest that A10-FMT could restore physiological tight-junction architecture, rectifying HFD-induced hyperexpression of barrier proteins. The selective efficacy of A10-FMT suggests microbiota-dependent modulation of renal epithelial integrity, thereby supporting physiological transmembrane transport processes and mitigating dysregulated electrolyte handling.

## 4 Discussion

Obesity-associated kidney injury is increasingly recognized as an important contributor to chronic kidney disease (CKD) and end-stage renal disease (ESRD). In addition to glomerular hyperfiltration and hemodynamic stress, obesity is commonly accompanied by dyslipidemia, oxidative stress, inflammation, and fibrosis, all of which contribute to progressive renal damage (7, 8, 10, 33, 44). Among these factors, renal lipotoxicity has emerged as a key pathogenic feature, as excessive lipid accumulation can directly impair renal cells and exacerbate kidney injury. Increasing evidence also supports an important role for the gut-kidney axis in obesity-related renal damage. High-fat diet (HFD)-induced disruption of intestinal homeostasis may alter microbial composition and metabolite production, impair barrier integrity, and facilitate the systemic dissemination of pro-inflammatory and metabolically harmful factors, thereby contributing to renal dysfunction (45). In the present study, using an HFD-induced obesity model, we found that transplantation of small intestinal contents from AOS-treated donors (A10-FMT) improved renal function and attenuated histopathological injury in recipient mice. Specifically, A10-FMT reduced serum blood urea nitrogen (BUN) and creatinine (CRE) levels and alleviated HFD-induced renal structural abnormalities, supporting a protective effect against obesity-associated renal injury (26, 27). These findings suggest that AOS-induced alterations in the intestinal environment are, at least in part, transferable and capable of conferring renoprotective features. Notably, donor mice in Experiment I were maintained under the same baseline dietary condition, whereas recipient mice in Experiment II were challenged with an HFD. This design minimized donor-side dietary variability and enabled assessment of whether AOS-conditioned intestinal contents could transfer protective features to obese recipients. At the same time, the transferred material did not fully reproduce the intestinal microenvironment of HFD-fed mice receiving AOS treatment. Accordingly, the present transplantation results should be interpreted as evidence that AOS-conditioned small intestinal contents can confer renoprotective features in HFD-fed mice, rather than as a direct reconstruction of the gut state under combined HFD and AOS exposure.

The improvement in renal injury was accompanied by partial correction of systemic metabolic disturbances. HFD markedly increased serum total cholesterol (TC) and triglyceride (TG) levels and reduced total antioxidant capacity (T-AOC), indicating substantial metabolic imbalance. In contrast, A10-FMT ameliorated these abnormalities, whereas Con-FMT showed weaker or less consistent effects. Given that metabolic overload and oxidative stress are well-established drivers of obesity-related kidney injury, these changes may have contributed to the observed renoprotection. Consistent with this interpretation, untargeted metabolomic analysis suggested that A10-FMT partially restored HFD-disrupted circulating metabolite profiles. In our previous study, A10-FMT improved the blood metabolome and increased eicosapentaenoic acid (EPA), a polyunsaturated fatty acid reported to protect renal function (15). EPA has been shown to alleviate renal lipotoxicity by restoring autophagic flux (34). In addition, several metabolites associated with renal and electrolyte homeostasis, including 18-hydroxycorticosterone, aldosterone, tetrahydrodeoxycorticosterone, tocotrienols, and creatine, were dysregulated by HFD but partially normalized after A10-FMT (46–50). These observations suggest that the beneficial effect of A10-FMT may be mediated, at least in part, through improvement of host metabolic status, although the specific contribution of individual metabolites remains to be determined. It is also notable that the magnitude of improvement was not uniform across all metabolic indices, which is not unexpected in a complex metabolic disease model in which different parameters may vary in sensitivity, response kinetics, and pathway dependence.

The gut microbiota data further support the involvement of the gut-kidney axis in this process. HFD markedly altered intestinal microbial composition and predicted microbial functions, whereas A10-FMT partially corrected these changes. Previous studies have shown that HFD-induced gut dysbiosis can impair host metabolic homeostasis, enhance inflammatory signaling, and promote tissue injury in distal organs. In this context, our findings suggest that AOS reshapes the intestinal environment in transferable manner that is associated with improved renal outcomes in recipient mice. Because the transplanted material consisted of small intestinal contents rather than defined bacterial strains, the protective effect is likely mediated by a combination of microorganisms, microbial metabolites, and other luminal factors. This point is important because it suggests that the renoprotective effect associated with AOS may not depend on a single microbial taxon, but rather on broader reprogramming of the gut ecosystem. However, this also means that the transplantation experiment should be interpreted cautiously, because it does not by itself establish a definitive causal role for specific bacteria or metabolites. Rather, it provides supportive evidence that gut-derived factors contribute to the renal benefits associated with AOS intervention.

At the renal molecular level, A10-FMT modulated several pathways relevant to kidney homeostasis. First, A10-FMT normalized HFD-associated alterations in the renal renin–angiotensin system (RAS). The RAS is a central regulator of renal hemodynamics as well as water and electrolyte balance, and its dysregulation contributes to obesity-associated renal injury (36). In the present study, HFD increased renal ANG II expression and decreased AGT and ATR1 levels, whereas A10-FMT partially restored these abnormalities (37, 38). Although the functional implications of these changes require further clarification, the present data suggest that gut-derived intervention may influence renal RAS-related signaling under HFD conditions.

A10-FMT was also associated with changes in proteins related to renal structural integrity and metabolic regulation. Specifically, HFD altered the expression of *α*-smooth muscle actin (α-SMA), ZG16, and CYP26B1, and these changes were partially reversed by A10-FMT (39, 51). In addition, HFD-induced abnormal expression of the junction-related proteins ZO-1, Claudin-1, Claudin-11, and E-cadherin was normalized after A10-FMT treatment (52–56). Because epithelial integrity and tissue structural homeostasis are essential for normal kidney function, these findings provide further evidence that A10-FMT exerts a broad protective effect on renal tissue organization in HFD-fed mice.

Inflammation is another major feature of obesity-related kidney injury. HFD is known to induce oxidative stress, macrophage infiltration, mitochondrial dysfunction, and activation of pro-apoptotic and pro-inflammatory pathways, thereby promoting lipid accumulation and renal damage (7, 11). These processes are often accompanied by increased inflammatory signaling in proximal tubular epithelial cells (PTECs) (4). Moreover, lipid disorder-associated inflammation is closely linked to the initiation and progression of CKD and is considered a potential therapeutic target in renal disease (4, 33, 57). In agreement with these reports, our RNA-seq analysis highlighted immune-related pathways among the major processes altered by HFD, and immunofluorescence analysis further showed that HFD increased renal TNF-α, TGF-β, and IL-22R expression. A10-FMT reduced all three markers, suggesting that attenuation of renal inflammation is likely an important component of the associated renoprotective effect. Taken together, these findings support the view that the protective effects associated with A10-FMT are not limited to a single pathway, but instead involve coordinated regulation of metabolism, inflammatory signaling, and renal structural homeostasis.

The present study has several strengths. Most importantly, it goes beyond demonstrating a direct protective effect of AOS by showing that the beneficial phenotype can be partially transferred through transplantation of small intestinal contents from AOS-treated donors. This design provides experimental support for the concept that gut-derived factors mediate, at least in part, the renal benefits of AOS. Several limitations should also be acknowledged. The transferred protective effect was demonstrated using whole small intestinal contents, and therefore the specific microbial or molecular mediators remain undefined. In addition, microbiota analysis was based mainly on 16S rRNA profiling and predictive functional inference, which limits taxonomic and mechanistic resolution. Although A10-FMT was associated with coordinated improvements in metabolic, inflammatory, and renal structural parameters, these findings do not by themselves establish direct causality. Moreover, the metabolomics results reflect changes in relative metabolite abundance and should be considered an initial characterization rather than a comprehensive pathway-level analysis. Future studies using defined microbial consortia, targeted metabolite validation, antibiotic depletion, or gnotobiotic models will be needed to identify the key mediators underlying these effects. In addition, translation of these findings to humans will require careful evaluation of safety, dosage, and long-term efficacy, particularly in individuals with metabolic syndrome or early-stage CKD.

## 5 Conclusion

In summary, our study demonstrates that A10-FMT partially attenuates HFD-induced renal dysfunction, histopathological injury, metabolic disturbance, and renal inflammation in mice. These results suggest that gut-derived factors contribute, at least in part, to the renoprotective effects of AOS. Collectively, our findings support a role for the gut-kidney axis in obesity-associated renal injury and provide a rationale for further investigation of intestinal environment-targeted strategies in the prevention and treatment of obesity-related kidney disease.

## Data Availability

The raw data analyzed in this study have been submitted to the NCBI database (accession numbers PRJNA734312 and GSE173566).

## Footnotes

1
www.biosys.bgi.com
